# Bst1 is required for *Candida albicans* infecting host via facilitating cell wall anchorage of Glycosylphosphatidyl inositol anchored proteins

**DOI:** 10.1038/srep34854

**Published:** 2016-10-06

**Authors:** Wei Liu, Zui Zou, Xin Huang, Hui Shen, Li Juan He, Si Min Chen, Li Ping Li, Lan Yan, Shi Qun Zhang, Jun Dong Zhang, Zheng Xu, Guo Tong Xu, Mao Mao An, Yuan Ying Jiang

**Affiliations:** 1 Shanghai Tenth People’s Hospital, and Department of Pharmacology, Tongji University School of Medicine, Shanghai, 200092, P.R. China; 2 Department of Anesthesiology, Changzheng Hospital, Second Military Medical University, Shanghai, 200433, P.R. China; 3 Department of dermatology, Shanghai Tongji Hospital, Tongji University School of Medicine, Shanghai, 200065, P.R. China; 4 Department of Laboratory Diagnosis, Changhai Hospital, Second Military Medical University, Shanghai 200433, P.R. China; 5 Research and Development Center of New Drug, School of Pharmacy, Second Military Medical University, Shanghai, 200433, P.R. China

## Abstract

Glycosylphosphatidyl inositol anchored proteins (GPI-APs) on fungal cell wall are essential for invasive infections. While the function of inositol deacylation of GPI-APs in mammalian cells has been previously characterized the impact of inositol deacylation in fungi and implications to host infection remains largely unexplored. Herein we describe our identification of *BST1*, an inositol deacylase of GPI-Aps in *Candida albicans*, was critical for GPI-APs cell wall attachment and host infection. *BST1*-deficient *C. albicans* (*bst1Δ/Δ*) was associated with severely impaired cell wall anchorage of GPI-APs and subsequen unmasked β-(1,3)-glucan. Consistent with the aberrant cell wall structures, *bst1Δ/Δ* strain did not display an invasive ability and could be recognized more efficiently by host immune systems. Moreover, *BST1* null mutants or those expressing Bst1 variants did not display inositol deacylation activity and exhibited severely attenuated virulence and reduced organic colonization in a murine systemic candidiasis model. Thus, Bst1 can facilitate cell wall anchorage of GPI-APs in *C. albicans* by inositol deacylation, and is critical for host invasion and immune escape.

*Candida albicans* is an opportunistic fungal pathogen that typically grows as a harmless commensal as a part of the normal flora found on the skin, mucosal surfaces, and in the gut of healthy individuals^1^. However, in immunocompromised populations, *C. albicans* infection can result in a diverse range from mild irritation to life-threatening systemic candidiasis. Importantly, despite significant medical advances, bloodstream infections of *C. albicans* are still associated with a high mortality rate^2^.

The fungal cell wall, as the outermost cellular structure, is a complex of cross-linked polysaccharides and glycoproteins only critical for the integrity and shape of fungi as they grow and differentiate, but also a key determinant of virulence. Polysaccharides such as β-glucans and mannans serve as pathogen-associated molecular patterns (PAMPs) that can be recognized by a variety of host-expressed pattern-recognition receptors (PRRs) including toll-like receptors (TLRs), nucleotide-oligomerization domain-like receptors (NLRs), and the recently identified family of spleen tyrosine kinase-coupled C-type lectin receptors (CLRs)^3^. PRRs recognition of PAMPs triggers an innate immune cell response and renders antigen presenting cells competent to prime T cells, ultimately resulting in activation of the adaptive immune system^4^.

Linked to polysaccharides are mannoproteins localizing on the outermost cell wall. As major covalently-linked mannoproteins, glycosylphosphatidyl inositol anchored proteins (GPI-APs) specifically attach to cell wall β-(1,6)-glucan through GPI remnant^5^. The GPI anchor is thus critical for targeting all these proteins to the cell wall. Previous studies have indicated that GPI-APs contribute to cell wall integrity, biofilm formation, adherence to host cells and abiotic medical devices, invasion of epithelial layers, and iron acquisition^6^. Notably, these studies highlighted the effects associated with deleting a GPI-AP, such as Ecm33p, specifically noting reduced virulence of fungi^7^. As such, it is not unexpected that deletions in the GPI biosynthetic pathway may block all GPI-APs cell wall attachments and thus be fatal for *C. albicans*. Taken together, these findings suggest that this biosynthetic pathway may represent a potential candidate target for antifungal agents.

GPI anchor is synthesized in the endoplasmic reticulum (ER) through the stepwise addition of sugars and ethanolaminephosphate (EtNP) to phosphatidylinositol^8^. In the early step of GPI biosynthesis in *C. albicans*, the inositol moiety is acylated by the action of Gwt1 (GPI-anchored wall protein transfer 1) protein to generate GlcN-(acyl) PI [glucosaminyl(acyl)phosphatidylinositol]. Inositol acylation is required for the addition of a terminal EtNP which links GPI to proteins. Recent studies have identified a novel antifungal candidate agent E1210, which targets inositol acylation to interrupt GPI biosynthesis^9^,^10^,^11^,^12^,^13^,^14^,^15^,^16^. Notably, when inositol acylation is defective, the cell surface expression of mammalian GPI-APs was also remarkably decreased^17^. The acyl-chain linked to inositol is removed by deacylation soon after the attachment of terminal EtNP to the proteins. However, *PGAP1* (Post GPI Attachment to Proteins 1) in mammalian cells [*BST1* (Bypass of Sec Thirteen 1) gene in yeast], acting as a inositol deacylase, is not required for the cell surface attachment of GPI-APs^18^,^19^. Therefore, there exists different roles for inositol acylation and deacylation on the cell surface expression of mammalian GPI-APs that may make inositol deacylation inhibition a superior antifungal strategy. Although inositol deacylase in *Saccharomyces cerevisiae* has been demonstrated^18^, its role in the transport and cell wall anchorage of GPI-APs remains unknown. In addition, the importance of inositol deacylation in the cell wall attachment of GPI-APs and host infection of pathogenic fungi has yet to be fully explored.

Herein the present study, we first demonstrated that Bst1 can facilitate GPI-APs targeting to cell wall by inositol deacylation in human pathogen *C. albicans*. Our results also demonstrate that *C. albicans* with defective inositol deacylase exhibit impaired invasive ability and enhanced recognition by host immune systems.

## Results

### Orf19.1053 (*BST1*) is responsible for inositol deacylase of GPI-APs in *C. albicans*

The sensitivity of GPI-APs to phosphoinositide-phospholipase C (PI-PLC) was used to evaluate the level of inositol deacylation in our study. After attaching to GPI anchors, GPI-APs are resistant to PI-PLC. Following inositol deacylation in the ER, GPI-APs become sensitive to PI-PLC and are transferred from the detergent phase to aqueous phase when separated by Triton X-114^18^.

**Figure 1:**
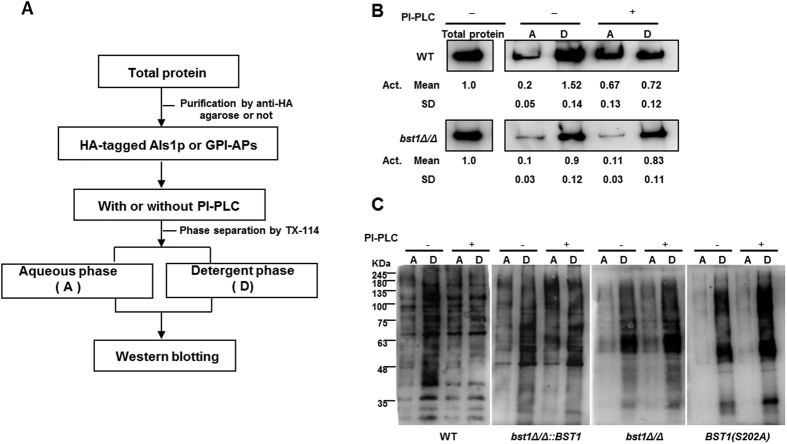
Defective inositol deacylation of GPI-APs in *BST1*-deficient *C. albicans* strains. (**A**) Flow diagram used to investigate the inositol deacylation of GPI anchored protein (Als1p) in *C. albicans* assessed by its sensitivity to bacterial phosphatidylinositol-specific phospholipase C (PI-PLC). (**B**) Sensitivity of Als1p from parent and *bst1Δ/Δ* mutant strains to PI-PLC. Purified HA-tagged Als1p were treated as specified in Fig. 1A. After phase separation, both aqueous (**A**) and detergent (**D**) phases of Als1p underwent immunoblotting using anti-HA tag antibody. (**C**) Sensitivity of mannoproteins from parent SN152, *BST1*-complemented, *bst1Δ/Δ* null mutant and *BST1*S202A mutant strains to PI-PLC. Cytoplasmic proteins from the detergent (**D**) phases were treated as specified in Fig. 1A. After phase separation, both aqueous (**A**) and detergent (**D**) phases of proteins underwent immunoblotting using 2.5 μg/ml peroxidase labeled-Con.

*C.albicans* expresses on its surface Als (Agglutinin like sequence) proteins, which play an important role in the development of candidiasis. Als1p, a well characterized GPI-anchored protein, is known to mediate the adhesion of *C. albicans* to host cells^20^. We extracted the hemagglutinin (HA)-tag fused Als1p from *bst1Δ/Δ* (*bst1Δ/Δ als1Δ/ALS1*-HA) and parent strain (*als1Δ/ALS1*-HA), and found that the majority of the purified Als1p from both strains were partitioned into the detergent phase. However, a subset of Als1p from the parental strain was partitioned into the aqueous phase following treatment with PI-PLC. By contrast, the majority of Als1p from *bst1Δ/Δ* strain remained partitioned into the detergent phase following PI-PLC treatment, suggesting that GPI-APs from *bst1Δ/Δ* strain were resistant to PI-PLC (Fig. 1B). This result indicated that inositol deacylation of GPI-APs was defective in *bst1Δ/Δ* mutant, and suggested that *BST1* played an important role in this process.

To further confirm the role of *BST1* in inositol deacylation of GPI-APs, we extracted cytoplasm proteins from *C. albicans* and demonstrated that GPI-APs with peroxidase labeled concanavalin A (ConA) can bind to mannose residues of GPI-anchor. ConA-stained GPI-APs from *bst1Δ/Δ* strain were found to be resistant to PI-PLC, as compared to the parental strain.

According to the sequence information from *Saccharomyces* genome database, the catalytic site of *BST1* in *S. cerevisiae* is serine-236. Meanwhile, the catalytic site of PGAP1 (homology gene of *BST1*) in human has been reported to be serine-174. The corresponding serine of *BST1* in *C. albicans* was found located in its 202 site by BLAST (basic local alignment search tool). Therefore, we mutated the serine-202 into alanine to ensure that the observed phenotypes are in fact due to the genetic defects at the *BST1* locus rather than artifacts from mutations accumulated during strain construction. We constructed a *C. albicans* strain [*BST1*(S202A)] in which the putative catalytic serine 202 of *BST1* was substituted for alanine, and found that its ConA-stained GPI-APs exhibited similar resistance to PI-PLC with *bst1* null mutant (Fig. 1C).

The above results indicate that *BST1* is responsible for inositol deacylation of GPI-APs in *C. albicans*.

### Impaired cell wall anchorage of GPI-APs in *BST1*-deficient *C. albicans* strains

GPI-APs link to the cell wall β-(1,6)-glucan through GPI remnant in *C. albicans* via phosphodiester bonds which can be cleaved using hydrogen fluoride (HF)-pyridine^5^,^21^. The highly mannosylated cell wall GPI-APs were extracted from *C. albicans* cells using HF-pyridine, and stained by peroxidase-linked ConA.

**Figure 2:**
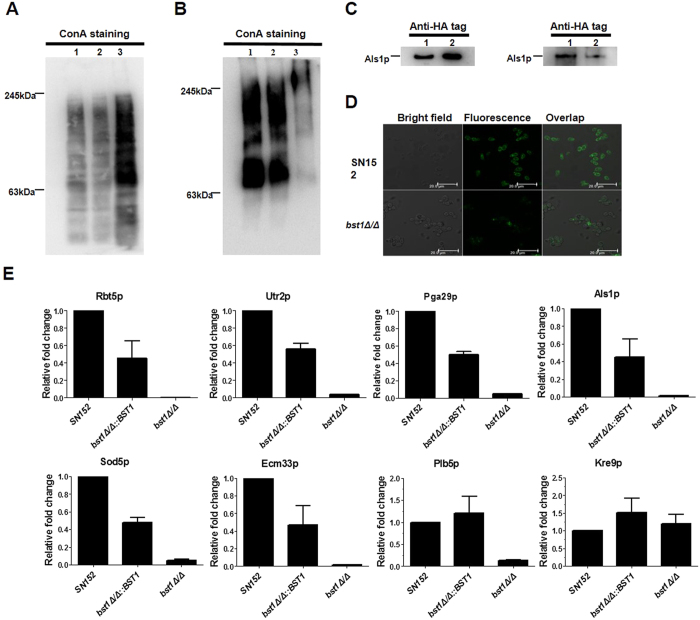
Cell wall anchorage of GPI-APs in *BST1*-deficient C. albicans strains was seriously impaired. (**A**) Intracellular GPI-APs from crude protein extracts prepared from exponentially growing parent strain (SN152) (Lane 1), *BST1*-complemented (*bst1Δ/Δ::BST1*) (Lane 2) and *bst1Δ/Δ* mutant strains (Lane 3) underwent immunoblotting using peroxidase labeled-ConA. (**B**) HF-released cell wall anchored GPI-Aps from cell wall debris of exponentially growing parent strain (SN152) (Lane 1), *BST1*-complemented (*bst1Δ/Δ::BST1*) (Lane 2) and *bst1Δ/Δ* mutant strains (Lane 3) underwent immunoblotting using peroxidase labeled-ConA. (**C**) Als1p extracted from crude protein extracts (left panel) or HF-treatment cell wall debris (right panel) of parent (*als1Δ/ALS1-HA*) (Lane 1), *bst1Δ/Δ* mutant strain (*bst1Δ/Δ als1Δ/ALS1-HA*) (Lane 2) using anti-HA tag antibody. (**D**) Fluorescence images exhibiting cell wall anchorage of Als1p in parent and *bst1Δ/Δ* mutant strains by confocal laser scanning microscope using anti-HA tag antibody. (**E**) Relative fold change of representative and virulence related GPI-APs with impaired cell wall anchorage in *BST1*-deficient *C. albicans* strains. The cell wall proteins of SN152, *bst1Δ/Δ::BST1*, and *bst1Δ/Δ* strains were analyzed by LC-MS/MS on high-resolution instruments (LTQ-Orbitrap XL and Velos, Thermo Fisher). Raw files were processed by MaxQuant (version 1.3.0.5) for peptide/protein identification and quantification.

Immunoblotting of cell wall GPI-APs extraction from parent strain with peroxidase labeled ConA revealed marked protein bands (Fig. 2B). while a remarkable reduction of ConA-stained material was observed in the *bst1Δ/Δ* strain, suggesting fewer GPI-APs linkage to the cell wall. In addition, we also found mannoproteins significantly accumulated in the cytoplasm of *bst1Δ/Δ* strain which was supported by an increase of ConA-stained materials (Fig. 2A).

Our results demonstrated that the strains expressing HA-tag fused Als1p (*bst1Δ/Δ als1Δ/ALS1-HA* and *als1Δ/ALS1-HA*) mediates adhesion similar to the untagged version (Data not shown). Immunoblotting of the cell wall HF-pyridine extraction from *C. albicans* strains expressing HA-tag fused Als1p (*bst1Δ/Δ als1Δ/ALS1*-HA; *als1Δ/ALS1*-HA) using an anti-HA-tag antibody revealed a reduction of Als1p in *bst1Δ/Δ* strain as compared to parent strain (Fig. 2C). A similar intracellular accumulation of Als1p was also observed in *bst1Δ/Δ* strain (Fig. 2C). Furthermore, we found a markedly reduced Als1p attachment to the cell wall of *bst1Δ/Δ* strain compared with parent strain (Fig. 2D) when evaluating these strains by immunofluorescence via an anti-HA antibody.

To further determine whether inositol deacylation was required for the cell anchorage of GPI-APs, we analyzed the cell wall HF-pyridine extraction by LC-MS/MS. Twenty-one GPI-APs were identified to be significantly reduced in the cell wall of *bst1Δ/Δ* strain as compared to the parent and *BST1*-complemented strains (see Supplementary Table S3). These proteins were thought to play specific roles, including participating in cell wall biogenesis and assembly (Pga4p, Cht2p, Utr2p, Dfg5p, Ecm33p, Dcw1p, Ccw14p, Crh11p and Ihd1p), adhesins and morphogenesis switch (Als1p, Pga24p and Pga29p), enzymes participating in host permitting their invasion and nutrient uptake (Plb5p, Carp9p, Csap and Rbt5p), degradation of damaging superoxide radicals (Sod4p and Sod5p), as well as other unknown functions (Pga30p, Pga45p, Pga52p)^6^. Seven of these proteins (Rbt5p, Utr2p, Pga29p, Als1p, Sod5p, Ecm33p and Plb5p) are known to impact the virulence of *C. albicans* (Fig. 2E)^7^,^20^,^22^,^23^,^24^,^25^,^26^.

### Inositol deacylation of GPI-APs is required for host invasion ability of *C. albicans*

GPI-APs of *C. albicans* participate in several stages of host invasive including morphogenesis (the reversible transition between unicellular yeast cells and hyphae), adhesion and invasion of host cells. Our results indicate a remarkable decreased in the attachment of GPI-APs to the cell wall of *bst1Δ/Δ*. Therefore, we assessed the invasion ability of the *bst1Δ/Δ* mutant strain *in vitro*.

**Figure 3:**
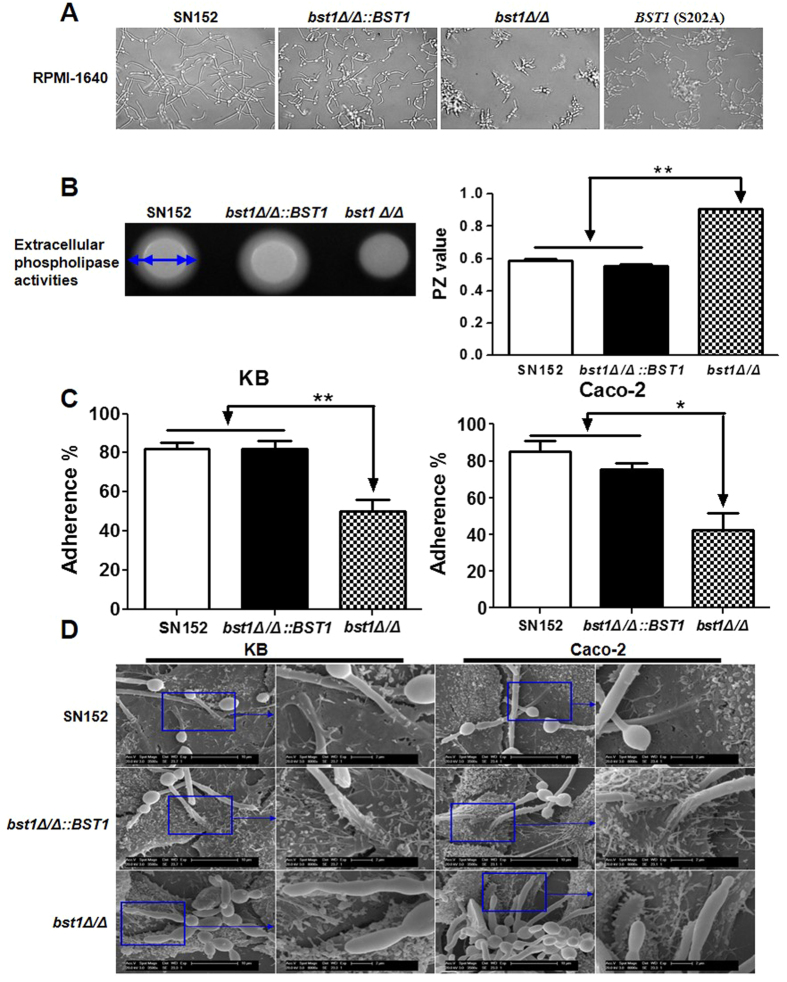
Deletion of *BST1* gene impairs the invasive ability of *C. albicans in vitro*. (**A**) Photomicrographs of parent (SN152), *BST1*-complemented (*bst1Δ/Δ::BST1*), *bst1Δ/Δ* null mutant (*bst1Δ/Δ*) and *BST1* S202A mutant strains growing in liquid RPMI 1640 culture at 37 °C for 3 hours to induce the hyphal form. (**B**) The total extracellular phospholipase activities of parent (SN152), *BST1*-complemented (*bst1Δ/Δ::BST1*), *bst1Δ/Δ* null mutant (*bst1Δ/Δ*) strains were determined by growing them on egg yolk agar at 37 °C for 2 days and measuring the precipitation zone around each colony (left panel). The phospholipase activity zone (PZ) values of all the strains were calculated by the ratio of colony diameter to diameter of the dense white zone of precipitation around positive colonies (right panel), data represent mean (±SD) of triplicates from one representative experiment of three. **P < 0.01 (Error bars indicate SD. One-way ANOVA with Bonferroni post-test). (**C**) The rates of adherence of parent (SN152), *BST1*-complemented (*bst1Δ/Δ::BST1*), *bst1Δ/Δ* null mutant (*bst1Δ/Δ*) strains to human KB oral epithelial cells or intestinal Caco-2 epithelial cells were evaluated by co-incubating them for 60 minutes in six-well tissue culture plates, after which the adherent clones were counted. Data represent mean (±SD) of triplicates from one representative experiment of three. **P < 0.01 (Error bars indicate SD. One-way ANOVA with Bonferroni post-test). (**D**) Representative micrographs of scanning electron microscope (SEM) of KB and Caco-2 cells invaded or penetrated by parent strain (SN152), *bst1Δ/Δ::BST1* and *bst1Δ/Δ* after 2 hours co-incubation.

*Bst1* mutants (*bst1Δ/Δ* and S202A mutant of *BST1*) displayed defective filamentation in either liquid (RPMI1640, 10% serum liquid medium) or solid media (Spider and Lee’s agar media) favoring hyphal growth (Fig. 3A and Supplementary Fig. S4). Our results indicate that the cell wall anchorage of phospholipase Plb5, which functions as an enzyme to damage the host cell membranes^26^, was decreased in *bst1Δ/Δ* mutant strain. So we assayed the total extracellular phospholipase activity by growing various strains on egg yolk agar and noted that *bst1Δ/Δ* exhibited significantly reduced secretion of phospholipases compared to the parent and *BST1*-complemented strains (Fig. 3B).

Adherence of *C. albicans* to epithelial cells, such as those in intestinal and oral mucosa, is essential for commensal growth and also an initial step in the pathophysiology of candidiasis. In the adherence assay, we found a significantly lower adherence, following a 60 minutes interaction period, of *bst1Δ/Δ* to KB (derived from a buccal carcinoma) and Caco-2 (derived from a colorectal carcinoma) monolayers as compared to the parent and *BST1*-complemented strains (Fig. 3C).

Following adhesion, the entry of *C. albicans* into host tissues is the next most crucial step in the pathophysiology of superficial and invasive candidiasis. We used a scanning electron microscopy (SEM) to visualize the invasion of *C. albicans* into KB and Caco-2 cells after 2 hours of co-culture. We observed the penetration of hyphal forms of the parent and *BST1*-complemented strains into KB and Caco-2 cells at the apical face, and microvillis were attached to their hyphae at the point of penetration (Fig. 3D). However, the *bst1Δ/Δ* mutant strain was not only defective in hyphal formation, but also hardly able to penetrate KB and Caco-2 cells (Fig. 3D).

These data suggest that diminished cell wall GPI-APs by *BST1* gene deletion in *C. albicans* significantly impairs its capacity to invade host cells.

### *BST1* is required for cell wall polysaccharides in *C. albicans*

**Figure 4:**
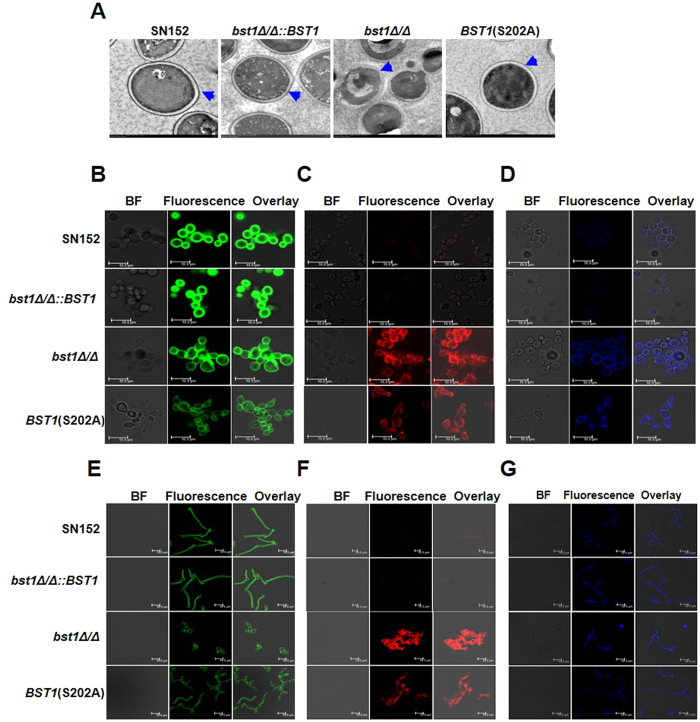
Deletion of *BST1* results in altered cell wall polysaccharides. (**A**) Representative micrographs of Transmission electron micrographs (TEM) of parent (SN152), *BST1*-complemented (*bst1Δ/Δ::BST1*), *bst1Δ/Δ* null mutant (*bst1Δ/Δ*), and *BST1* S202A strains to evaluate their ultrastructure of cell wall. Blue arrows indicate the outer surface mannoprotein fibrils of the cell walls. (**B–D**) Fluorescence micrographs of three cell wall carbohydrate layers from exponentially growing parent (SN152), *BST1*-complemented (*bst1Δ/Δ::BST1*), *bst1Δ/Δ* null mutant (*bst1Δ/Δ*), and *BST1* S202A strains, which were stained with ConA-FITC to visualize mannan (**B**), β-glucan antibody to visualize β-glucan (**C**) and CFW to visualize chitin (**D**). Scale bar represents 10 μm. (**E–G**) Fluorescence micrographs of three cell wall carbohydrate layers from the hyphal forms of parent (SN152), *BST1*-complemented (*bst1Δ/Δ::BST1*), *bst1Δ/Δ* null mutant (*bst1Δ/Δ*), and *BST1* S202A mutant strains (*Bst1* mutants displayed defective filamentation), which were stained with ConA-FITC to visualize mannan (**E**), β-glucan antibody to visualize β-glucan (**F**) and CFW to visualize chitin (**G**). Scale bar represents 10 μm.

The cell wall of *C. albicans* is composed of an inner skeletal layer of chitin and β-glucans (β-1,3- and β-1,6-glucan) and an outer layer of highly glycosylated proteins containing GPI-APs. Via transmission electron microscopy (TEM), we found that the inner cell wall layer of the parental strain is surrounded by an external layer of mannosylated proteins, while the *bst1Δ/Δ* strain was nearly devoid of an outer cell wall layer (Fig. 4A).

To further examine the effect of *BST1* deficiency on cell wall polysaccharides, we stained polysaccharides (mannan, β-glucan or chitin) with ConA alexa fluor 488 conjugate, anti-β-glucan primary antibody and calcofluor white (CFW). ConA alexa fluor 488 conjugate staining indicated that *bst1* mutants (*bst1Δ/Δ* and S202A mutant of *BST1*), either in yeast or hyphae form, had a markedly decreased mannan content within their cell walls (Fig. 4B,E and Supplementary Fig. S5A,D). β-(1,3)-glucan, a well characterized PAMP of *C. albicans*, is usually buried beneat the outer layer of highly glycosylated proteins. We determined the cell surface β-glucans content by anti-β-glucan staining, and found a marked exposure of β-(1,3)-glucans with significantly higher fluorescence intensity on the yeast or hyphae cell surface of *bst1* mutants as compared to the parent strain (Fig. 4C,F and Supplementary Fig. S5B,E). Meanwhile, an increase of CFW binding with significantly higher fluorescence intensity, indicating higher chitin content, was also observed in *bst1* mutants (Fig. 4D,G and Supplementary Fig. S5C,F).

Consistent with the aberrant cell wall structures, surface properties of the *bst1Δ/Δ* strain, such as hydrophobicity and flocculation, were also markedly increased (see Supplementary Fig. S6A,B). The change of cell wall structures and properties also impaired the resistance of *bst1Δ/Δ* to different stresses including oxidative stress, congo red, CFW, and antifungal agents (fluconazole and miconazole) (see Supplementary Fig. S6C).

### *C. albicans* with defective inositol deacylation of GPI-APs can induce stronger inflammatory response

**Figure 5:**
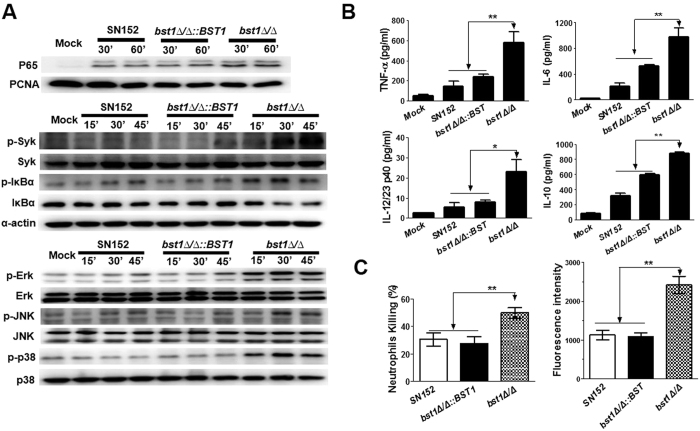
*BST1*-deficient *C. albicans* could be recognized by host innate immune cells. (**A**) *Bst1Δ/Δ* mutant strain induced NF-κB and MAPK activation and inflammatory responses in macrophages. Thioglycollate-elicited peritoneal macrophages were stimulated by UV-inactivated *C. albicans* yeast SN152, *bst1Δ/Δ*::*BST1*, *bst1Δ/Δ* (MOI = 5) for the indicated times. The nuclear extracts (top panel) and cell lysates (middle and lower panel) were analyzed by immunoblotting with the indicated antibodies. Mock, unstimulated macrophages. (**B**) ELISA of TNF-α, IL-6, IL-12/23p40, and IL-10 collected from the supernatants of macrophages challenged with UV-inactivated parent (SN152), *BST1*-complemented (*bst1Δ/Δ::BST1*), and *bst1Δ/Δ* null mutant strains for 6 hours. Data represent mean (±SD) of triplicates from one representative experiment of three. *P < 0.05; **P < 0.01 (One-way ANOVA with Bonferroni post-test). (**C**) Neutrophils killing rates of parent (SN152), *BST1*-complemented (*bst1Δ/Δ::BST1*), *bst1Δ/Δ* null mutant strains determined by incubation them with thioglycollate-elicited neutrophils for 1 hour, and the survival of the *C. albicans* were counted (left panel). Respiratory burst of neutrophils stimulated by the indicated *C. albicans* were assayed by detecting the cellular reactive oxygen species production in neutrophils after incubation with *C. albicans* for 1 hour (right panel). Data represent mean (±SD) of triplicates from one representative experiment of three. *P < 0.05 and **P < 0.01 (One-way ANOVA with Bonferroni post-test).

Macrophages are essential to initiate antifungal innate immune responses and also prime an adaptive immune response. We tested whether the *bst1Δ/Δ* mutant strain, could stimulate enhanced macrophage response. We found that *bst1Δ/Δ* mutant stimulation induced more nuclear translocation of nuclear factor-κB (NF-κB) (p65), spleen tyrosine kinase (Syk) phosphorylation, and inhibitor of kappa B α (IκBα) phosphorylation and degradation in thioglycollate-elicited peritoneal macrophages, suggesting increased NF-κB signaling. Consistent with this hypothesis, we observed enhanced extracellular-signal-regulated kinase (ERK), c-Jun N-terminal kinase (JNK) and p38 phosphorylations induced in macrophages by *bst1Δ/Δ* mutant strain stimulation as compared to the parental strain. These results confirm the activation of mitogen-activated protein kinase (MAPK) signaling (Fig. 5A). NF-κB and MAPK activation can modulate the expression of various inflammatory cytokines. We found that *bst1Δ/Δ* mutant strain induced higher levels of inflammatory cytokines including tumor necrosis factor α (TNF-α), interleukin 6 (IL-6), interleukin-12 p40 (IL-12p40), and interleukin 10 (IL-10) in macrophages, while the production of these cytokines were significantly lower in the parent and *BST1*-complemented strain, respectively (Fig. 5B). In addition, *BST1* (S202A) mutant strain also induced higher levels of inflammatory cytokines in macrophages (see Supplementary Fig. S7). We also noted that a higher percentage of *bst1Δ/Δ* strains were killed by neutrophils than those levels observed in the parent or *BST1*-complemented strains in co-culture model system. These results are consistent with an augmented respiratory burst response to *bst1Δ/Δ* strain (Fig. 5C).

**Figure 6:**
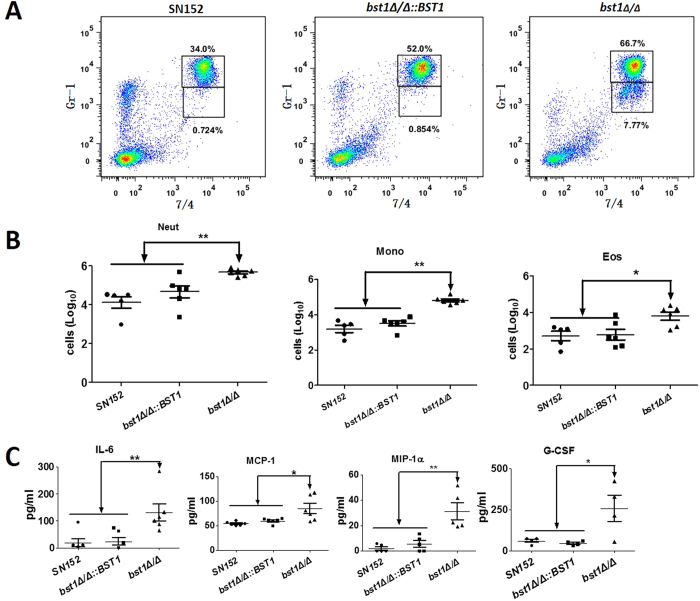
Enhanced *in vivo* inflammatory response to *C. albicans* by *bst1* deficiency. (**A**) Flow cytometry for Gr-1^hi^7/4^hi^ neutrophils and Gr-1^+^ 7/4^hi^ inflammatory monocytes in mice 4 hours following intraperitoneal infection with 5 × 10^5^ UV-inactivated yeast phase parent (SN152), *BST1*-complemented (*bst1Δ/Δ::BST1*), *bst1Δ/Δ* null mutant strains. Numbers adjacent to outlined areas indicate the percentage of neutrophils (top panel) and monocytes (lower panel). Data are representative images of 5 mice. (**B**) Scatter plots of myeloid cell subsets in the peritoneal cavities of mice after 4 hours of intraperitoneal infection with 5 × 10^5^ UV-inactivated yeast phase parent (SN152), *BST1*-complemented (*bst1Δ/Δ::BST1*), *bst1Δ/Δ* null mutant strains. Each symbol represents an individual mouse. Data are representative of three independent experiments.*P < 0.05; **P < 0.01 (Kruskal-Wallis nonparametric One-way ANOVA with Dunns post-test). (**C**) ELISA for cytokines, chemokines and growth factors in lavage fluid from the inflamed peritoneal cavities of mice after 4 hours of intraperitoneal infection with 5 × 10^5^ UV-inactivated yeast phase parent (SN152), *BST1*-complemented (*bst1Δ/Δ::BST1*), *bst1Δ/Δ* null mutant strains. IL-6, MCP-1, MIP-1α, G-CSF, GM-CSF. Data are representative of three independent experiments. *P < 0.05; **P < 0.01 (Kruskal-Wallis nonparametric One-way ANOVA with Dunns post-test).

We further explored how the aberrant cell wall structures of *bst1Δ/Δ* strain affected inflammation response *in vivo* using a peritoneal infection model^27^. After 4 hours of intraperitoneal infection with 5 × 10^5^ *C. albicans*, we found that *bst1Δ/Δ* mutant-infected mice had recruited more cells than mice infected with the parent strain, including Gr-1^hi^7/4^hi^ neutrophils, Gr-1^+^ 7/4^hi^F4/80^+^ inflammatory monocytes and Gr-1^int^7/4^lo^F4/80^+^ side-scatter-high eosinophils in the peritoneum (Fig. 6A,B). The enhanced inflammatory cell recruitment was also associated with increased production of specific cytokines and growth factors including IL-6, monocyte chemotactic protein-1 (MCP-1), macrophage inflammatory protein 1 α (MIP-1 α), granulocyte colony stimulating factor (G-CSF), and granulocyte-monocyte colony-stimulating factors (GM-CSF) (Fig. 6C). Thus, *C. albicans* with defective inositol deacylation of GPI-APs can induce stronger inflammatory response *in vivo*.

### Inositol deacylation of GPI-APs is required for invasive infection in a mouse model of hematogenously disseminated candidiasis

**Figure 7:**
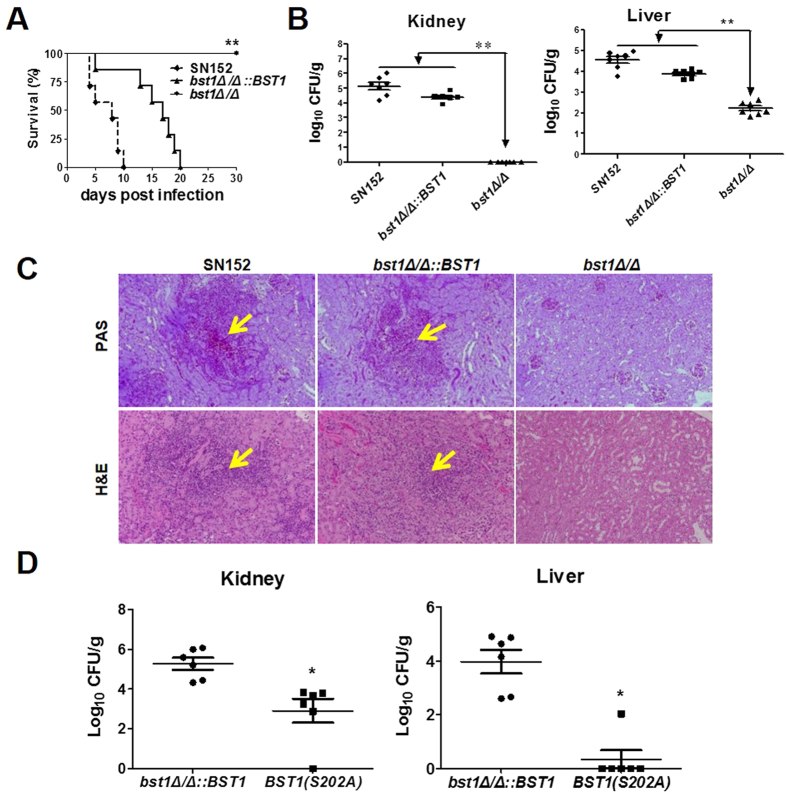
GPI inositol deacylation is required for invasive infection during hematogenously disseminated candidiasis. (**A**) Survival curves of thirty C57BL/6 female mice infected via the tail vein with 5 × 10^5^ CFU parent (SN152), *BST1*-complemented (*bst1Δ/Δ::BST1*), *bst1Δ/Δ* null mutant strains. Infected animals were observed for over 30 days. Data are representative of two independent experiments. **P < 0.01, (Log-rank test). (**B**) Quantification of the fungal cells in the livers and kidneys of mice intravenously infected with 5 × 10^5^ CFU parent (SN152), *BST1*-complemented (*bst1Δ/Δ::BST1*), *bst1Δ/Δ* null mutant strains. After two days of infection, tissues were isolated and homogenized, and CFUs were quantitated and normalized with respect to the tissue weight. Data are representative of two independent experiments. **P < 0.01 (Kruskal-Wallis nonparametric One-way ANOVA with Dunns post-test). (**C**) Kidney histopathology was analyzed with periodic acid-Schiff (PAS) and hematoxylin and eosin (H&E) staining at day 2 after intravenous infection with 5 × 10^5^ CFU parent (SN152), *BST1*-complemented (*bst1Δ/Δ::BST1*), *bst1Δ/Δ* null mutant strains. (**D**) Quantification of the fungal cells in livers and kidneys of mice intravenously infected with 5 × 10^5^ CFU *BST1*-complemented (*bst1Δ/Δ::BST1*) and *BST1S202A* mutant strains. After 5 days of infection, tissues were isolated and homogenized, and CFUs were quantitated and normalized with respect to tissue weight. Data are representative of two independent experiments. *P < 0.05 (Kruskal-Wallis nonparametric One-way ANOVA with Dunns post-test).

The dimorphism of *C. albicans* and its capabilities of adhesion and invasion of host epithelial cells are required for hematogenously disseminated candidiasis. The host immune response also significantly affects the outcome of invasive infection. Therefore, we investigated the contribution of *BST1* to the development of hematogenously disseminated candidiasis in a mouse model. Over a 30-day observation period, none of the mice infected intravenously with the *bst1Δ/Δ* mutant strains died (Fig. 7A). By contrast, the median survival of mice infected with parent and *bst1Δ/Δ::BST1*-complemented strains was 8 and 17 days, respectively (Fig. 7A). The kidney and liver fungal burdens of mice inoculated with *bst1* mutants (*bst1Δ/Δ* and S202A mutant of *BST1*) were significantly lower than those infected with either the parent or *BST1*-complemented strains (Fig. 7B,D).

The lower fungal burden in the kidneys of mice infected with the *bst1Δ/Δ* mutant strain was confirmed by histopathological review. Following two days of infection, no *C. albicans* filaments, except for a few inflammatory cells, were observed in the kidneys, of mice infected with the *bst1Δ/Δ* mutant strain (Fig. 7C). By contrast, in the parent or BST1-complemented strains, microabscesses were observed in the kidneys, which consisted of numerous neutrophils surrounding multiple *C. albicans* filaments. These results suggest that inositol deacylation of GPI-APs by *BST1* is required maximal virulence of *C. albicans* in the mouse model of hematogenously disseminated candidiasis.

## Discussion

The biosynthesis of GPI-APs can be separated into two phases. In the first phase, the GPI-anchor is synthesized and attached to the proteins. The inositol moiety of GPI is acylated at an early step in GPI biosynthesis, which is essential for the generation of mature GPI capable of proteins attachment in fungi. In the second phase, both the GPI and protein portions of the precursor GPI-APs are modified during transport from the ER to the cell surface^8^. The acyl group is usually absent from GPI-APs on the cell surface due to inositol deacylation that occurs in the ER shortly after GPI-anchor attachment in the second phase of GPI-APs biosynthesis. Mammalian inositol deacylase (Pgap1p) has been cloned and demonstrated to be important for efficient transport of GPI-APs from the ER to the Golgi, but not for the normal cell surface expression^18^,^19^. In the present study, we found that Bst1p could work as inositol deacylase in *C. albicans*, which was involved in the start of the second phase of GPI-APs biosynthesis. We also demonstrated that inositol deacylation of GPI-APs played a critical role in the pathogenicity of *C. albicans*.

Moreover, we demonstrated that GPI-APs from *bst1* mutant strain were more resistant to PI-PLC compared to those of parent strain, highlighting the function of Bst1p as inositol deacylase in *C. albicans* (Fig. 1B,C). Nevertheless, the remove of palmitate residue by inositol deacylase in many mammalian GPI-APs is not obligatory^28^. By example, the mature GPI-APs on human erythrocyte membrane remain an acylated status. Blocking inositol deacylase of GPI-APs via *PGAP1* deficiency in mammalian cells failed to affect the cell surface expression of GPI-APs, and only led to the expression of structurally abnormal GPI-APs with the acylated inositol^19^. However, our results suggested that inositol deacylation of GPI-APs was critical for their efficient anchorage to the cell wall of *C. albicans* (Fig. 2B–E). Consistent with the diminished cell wall GPI-APs, *bst1* mutant strains exhibited an accumulation of GPI-APs within the cytoplasm (Fig. 2A,C), indicating the key role of inositol deacylation in targeting GPI-AP to the cell wall. The different roles of inositol deacylation of GPI-APs in *C. albicans* and mammalian cells may be associated with the evolution of Bst1p. The evolutionary conservation of this protein is relatively low between *C. albicans* and mammalian cells, with a sequence similarity of only 28% (see Supplementary Fig. S8).

GPI-APs covalently link to the skeletal polysaccharides of fungal cell wall by GPI remnant. Many cell wall GPI-APs, such as Als1p, Als3p, Ecm33p and Hwp1p, act as virulence factors in the development of an invasive infection. In this work, we demonstrated that inositol deacylation is required for GPI-AP anchorage to the cell wall. Proteomic analysis indicated that GPI-APs including several known virulence factors diminished in the cell wall of *bst1Δ/Δ* strain. These virulence factors play roles in cell wall biogenesis and assembly (Utr2p, Ecm33p), adhesion, morphological switch (Als1p), invasion and damage to the host cells (Plb5p, Rbt5p), as well as degradation superoxide radicals to protect themselves (Sod5p) (see Supplementary Table S3). Consistent with the reduced cell wall virulence factor, *bst1Δ/Δ* mutant strain demonstrated defective abilities in the key steps of invasive infection including filaments transition, phospholipases secretion, adherence and invasion of host epithelial cells, which emphasized the importance of inositol deacylase (Fig. 3).

*C. albicans* cell wall is a complex and dynamic architecture based on a core structure of β-(1,3)-glucan covalently linked to β-(1,6)-glucan and chitin, as well as an outer layer or matrix composed mainly of mannose-glycosylated proteins^29^. These polysaccharides serve as PAMPs that can be recognized by PRRs which may subsequently trigger innate immune cell response and render antigen-presenting cells competent to prime T cells, thereby initiate the adaptive immunity. The recognition of β-(1,3)-glucan by Dectin-1 plays a crucial role in host antifungal defense. Previous studies have indicated that the Y238X polymorphism in Dectin-1 was associated with human mucosal *C. albicans* infection^30^. However, β-(1,3)-glucan is usually buried beneath the outer layer of mannoproteins except for the region between the parent cell and the mature bud, in which Dectin-1 is bound in a more restricted pattern. It has also been reported that exposure of cell wall β-(1,3)-glucan in *C. albicans* could increase its recognition by phagocytic cells^31^. Moreover, deficient inositol deacylase of GPI-APs in *C. albicans* could also induce β-(1,3)-glucan exposure (Fig. 4C,F). This exposure may be a result of the inadequate concealment of the inner layer by cell surface mannose-glycosylated proteins of the *bst1Δ/Δ* mutant strain.

Our study demonstrated that *bst1Δ/Δ* mutant strain could be recognized by the host innate immune cells and thus induced stronger immune responses. Neutrophils contribute to the initial response against fungi, and play a critically important role in individuals who are neutropenic or immunosuppressed^32^,^33^,^34^. We demonstrated that neutrophils could kill *bst1Δ/Δ* mutant strains more efficiently, and produced more ROS when challenged (Fig. 5C). After encountering microbial pathogens, host macrophages secrete cytokines and chemokines and engulf the pathogens in phagosomes^35^. Not only did *bst1Δ/Δ* mutant strains elicited an enhanced recognition by macrophages, but an increased cellular responses of macrophages was observed. This response included Syk phosphorylation, activation of NF-κB and MAPK signaling, and triggering cytokine production (TNF-α, IL-6, IL-12p40, and IL-10) (Fig. 5A,B). IL-6 and IL-23 (consists of IL-12 p40 and p19) are key cytokines leading to the induction of Th17 cell differentiation^36^. TNF-α and IL-6 participate in the innate immune response against *Candida* infection by promoting neutrophils production and activation. Increased levels of these inflammatory cytokines may result in a recruitment of innate immune cells such as neutrophils to the infected organs to clear fungi. Using a peritoneal infection model, we demonstrated that the marked elevation of chemokines in peritoneal cavity stimulated by *bst1Δ/Δ* mutant strain resulted in an obvious recruitment of neutrophils and monocytes (Fig. 6). Besides β-(1,3)-glucan recognition by Dectin-1, mannan recognition by Dectin-2/Dectin-3 is important in resistance to fungal infection^27^,^37^,^38^. The present study indicated that deletion of *BST1* in *C. albicans* resulted in decreased mannose content and a more remarkable exposure of β-(1,3)-glucan on the cell surface. Based on our present result, it could not determine that the change of mannan or exposure of β-(1,3)-glucan contribute to the altered host immune responses. The role of mannan recognition and glucan recognition in the altered immune responses induced by *bst1* mutant is worth to be further investigated.

The development of invasive fungal infection is determined both by the invasive ability of the infective strains and the host antifungal defense. Our results suggested that defective inositol deacylation of GPI-APs not only inhibited the invasive ability of *C. albicans*, but also enhanced its recognition by the host immune system. As expected, our study further demonstrated that *bst1Δ/Δ* mutant strain displays a markedly diminished virulence in hematogenously disseminated candidiasis (Fig. 7). Both impaired invasive ability and enhanced immunogenicity of *bst1Δ/Δ* mutant strain contribute to its diminished virulence. We demonstrated the effects on virulence are not a result of a reduce growth rate in the *bst1* null mutant (see Supplementary Fig. S9).

In conclusion, our study describes the novel efffets of Bst1 acting as inositol deacylase of GPI-APs in *C. albicans*, and facilitating cell wall anchorage of GPI-APs. Our study suggests that abolishing inositol deacylation of GPI-APs in *C. albicans* not only impaired its invasion ability, but also exposed PAMPs recognized by host immune system. Furthermore, inositol deacylation of GPI-APs defection rendered *C. albicans* more sensitive to azole antifungal agents. Therefore, our findings may provide a potential target for the development of new drugs with a new modality of treatment as compared to current antifungal agents. Given the high mortality assoiated with systemic candidiasis infection, future studies should urgently evaluate the crystal structure of Bst1p in an effort to identify potential drug candidates by high-throughput screening.

## Methods

### Ethics statement

All mouse experimental procedures were performed in accordance with the Regulations for the Administration of Affairs Concerning Experimental Animals approved by the State Council of People’s Republic of China. The protocol was approved by the Institutional Animal Care and Use Committee of Tongji University.

### Strain construction and growth conditions

All strains were maintained on SDA agar (1% peptone, 4% dextrose, and 1.8% agar) plates. For *in vitro* experiments, strains were grown in YPD broth overnight in a rotary shaker at 30 °C. For hyphae growth, 1 × 10^6^ *C. albicans* were cultured in RPMI 1640 medium [RPMI 1640 (Gibco BRL) 10 g, NaHCO_3_ 2.0 g and morpholinepropanesulfonic acid (Sigma) 34.5 g, pH 7.0, in 1 L ddH_2_O, sterilization by filtration] at 37 °C for 3 hours.

The entire coding sequence of *BST1* was deleted from the parent strain SN152 by two-step homologous recombination using a fusion-PCR-based strategy as described previously^39^ (see Supplementary Fig. S1). The *BST1*-complemented (*bst1Δ/Δ:: BST1*) and *BST1* S202A mutant strain were generated using the *SAT1* flipping method^40^,^41^ (see Supplementary Figs S1 and S2). We got 8 independently created homozygous *bst1* mutants of *C. albicans* totally. These homozygous mutants (Mutant 1#, 2#, 3#, 4#, 5#, 6#, 7#, 8#) showed similar phenotype (Data not shown), and we showed the phenotype of mutant 1# in our manuscript.

Strains expressing HA-tag fused Als1p (*bst1Δ/Δ als1Δ/ALS1-HA* and *als1Δ/ALS1-HA*) were generated in strain *bst1Δ/Δ* and SN152. One allele of *ALS1* was deleted according to the fusion-PCR-based strategy, and a 102-bp fragment containing three repeated sequence of HA epitope-tag was inserted into the *ALS1* gene allele using the *SAT1* flipping method (see Supplementary Fig. S3). And we validated the tagging by sequencing of the mutant.

All of the strains and primers used for strain construction are listed in Supplementary Tables S1 and S2.

### PI-PLC sensitivity assay

The sensitivity of GPI-APs to PI-PLC was used to evaluate the level of inositol deacylation. PI-PLC sensitivity assay was performed as described previously with some modifications^42^. HA-tag fused Als1p was purified by anti-HA-tag mouse mAb (agarose conjugated) from *C. albicans* expressing HA-tag fused Als1p. The purified Als1p was incubated with or without 0.5 unit of PI-PLC for 1h at 30 °C and then separated into aqueous and detergent phase by 1.4% Triton X-114. The aqueous and detergent phase were subjected to SDS-PAGE, and then blotted using anti-HA monoclonal antibody. Cytoplasm proteins were partitioned into aqueous and detergent phase using Triton X-114. The detergent phase were collected and incubated with 1 unit of PI-PLC for 1h before phase separation. Both phases were analyzed by western blotting analysis using 2.5 μg/ml peroxidase labeled-ConA.

### Isolation and analysis of CWPs and cytoplasmic proteins

Covalently-bound CWPs were extracted by HF-pyridine, as previously described^20^,^43^,^44^. Cytoplasmic proteins were extracted by cell lysis buffer (50 mM Tris-HCl, PH7.4, 150 mM NaCl, 5 mM EDTA, 1 mM PMSF and protease inhibitors). CWPs and cytoplasmic proteins were analyzed by immunoblotting with 2.5 μg/ml peroxidase labeled-ConA respectively. The CWPs were trypsin-digested and analyzed by LCMS/MS on a high-resolution instrument (LTQ-Orbitrap XL and Velos, Thermo Fisher). MaxQuant (version 1.3.0.5) was used for peptide/protein identification and quantification.

*C. albicans* expressing HA-tag fused Als1p (*bst1Δ/Δ als1Δ/ALS1-HA* and *als1Δ/ALS1-HA*) were stained with anti-HA-tag antibody followed by Alexa-488-labeled goat anti-mouse antibody, and scanned under an oil immersion objective at 63× magnification on a confocal microscope (TCS SP5; Leica).

### Cell wall integrity assays

*C. albicans* strains were grown in YPD broth in a rotary shaker at 30 °C, and exponentially growing *C. albicans* yeast were harvest. For hyphae growth in our study, 1 × 10^6^ *C. albicans* were cultured in RPMI 1640 medium [RPMI 1640 (Gibco BRL) 10 g, NaHCO_3_ 2.0 g and morpholinepropanesulfonic acid (Sigma) 34.5 g, pH 7.0, in 1 L ddH_2_O, sterilization by filtration] at 37 °C for 3 hours. And then the hyphae cells were harvest. *C. albicans* yeast or hyphae cells were then stained with specific anti-β-glucan primary antibody overnight at 4 °C on a rotator and Cy3-labeled goat-anti mouse secondary antibody for 1 h at 30 °C to visualize β-glucan exposure^45^. To stain the cell wall for chitin and mannan, cells were incubated with 50 μg/ml CFW or 50 μg/ml ConA alexa fluor 488 conjugate for 45 minutes away from light^46^. The stained yeast or hyphae cells described above were scanned with a confocal laser scanning microscope (TCS SP5; Leica). The fluorescence intensity of confocal laser scanning microscope photograph for Fig. 4B–G were acquired and analyzed by LAS AF Lite program (Version 2.1.1 build 4443, Leica Microsystems) (n = 100 for yeast cells and n = 20 for hyphae).

The ultrastructure of cell wall was observed by TEM. Exponentially growing *C. albicans* were washed in PBS and fixed in 3ml fixative solution (sodium cacodylate buffer, pH 7.2, containing 4% polyoxymethylene) for 24 h at 4 °C. The samples were then washed with saline and postfixed with 1% phosphotungstic acid for 90 minutes. The fixed cells were dehydrated increasing concentrations of ethanol. Ultrathin sections were prepared, double stained with uranium and lead, and observed under a transmission electron microscope (Hitachi H-800, Japan).

### Murine systemic candidiasis model

C57BL/6 female mice (6–8weeks) were anaesthetized intraperitoneally before infection. A total of 5 × 10^5^ live *C. albicans* strains were administrated intravenously in a final volume of 200 μl of saline. Survival rates were monitored for 30 days. Fungal burden was assessed by plating a series of diluted solutions of homogenized kidneys and livers from mice at 2 days or 5 days after infection. For histopathological analysis, kidneys were fixed in 10% neutral buffered formalin and stained with hematoxylin and eosin (HE) and periodic acid-schiff (PAS) to reveal inflammatory infiltration and fungal hyphae.

### Phospholipase activity assays of *C. albicans*

The phospholipase activity was assayed according to the method previously described^47^. A total of 10^5^ exponentially growing *C. albicans* in PBS was plated on egg yolk agar (consisting of Sabouraud’s dextrose agar, NaCl, CaCl _2_ and egg yolk emulsion) and incubated at room temperature for 2 days. The extracellular phospholipase of each strain was determined by measuring the width of zone of precipitation around the colony.

### Epithelial cell adherence and invasion assay

Adherence assays were performed, according to a previously described protocol, using two cell lines^48^. Caco-2 cells and KB cells were grown to 95% confluency in a 6-well tissue culture plate. Each well was infected with 10^2^ organisms. After 60 minutes of infection, the wells were then overlaid with 2 ml of YPD agar (about 45 °C) and incubated at 30 °C for 48 hours and the percentage of adherence was analyzed.

Caco-2 and KB cells were grown to 95% confluency on 8 mm diameter glass coverslips. Each coverslip was infected with 1 × 10^6^ exponentially growing *C. albicans*. After 2 hours of infection, the epithelial cells were examined using a XL-30 SEM (Philips, Holland)^49^.

### Thioglycollate-elicited peritoneal macrophage and neutrophil preparation

C57BL/6 mice were injected intraperitoneally with 2 ml of 3% (weight/volume) thioglycollate (Merck). Peritoneal cells were collected by washes with PBS containing 0.5 mM EDTA at 14 and 72 hours, respectively, for neutrophil and macrophage isolation, respectively^27^. The cells were cultured in RPMI1640 plus 10% heat-inactivated fetal bovine serum (volume/volume).

### Macrophage-yeast interaction

*C. albicans* yeast cells were harvested, and exposed to four doses of 100,000 μjoules/cm^2^ in a CL-1000 Ultraviolet Crosslinker (UVP), with agitation between each dose to treat cells evenly^50^. The thioglycollate-elicited macrophages were stimulated with the UV-inactivated *C. albicans* [multiplicity of infection (MOI) = 5] for the indicated time.

For nuclear extracts, 1 × 10^7^ murine macrophages were incubated in 6 cm plates and stimulated with UV-inactivated *C. albicans* for 30 minutes and 1 hour, respectively; whereas for total cell lysates, 3 × 10^6^ cells were incubated in 12-well plates and stimulated with UV-inactivated *C. albicans* for 15, 30, and 45 minutes, respectively. For cytokine production assay, 1 × 10^7^ cells were incubated in 6-well plates and stimulated with the UV-inactivated *C. albicans* for 6 hours, the cell supernatants were collected for cytokine production assay.

### Western blotting

For total cell lysates, cells were lysed in lysis buffer (250 mM NaCl, 50 mM HEPES, 1 mM EDTA, 1% Nonidet P-40, 1 mM Na_3_VO_4_, 1 mM NaF, and protease inhibitors, pH = 7.4). For nuclear extracts, cells were lysed in lysis buffer (10 mM HEPES, 10 mM KCl, 1.5 mM MgCl_2_, 0.5 mM DTT, 0.05% Nonidet P-40, and protease inhibitors, pH = 7.9), and extracted in extraction buffer (5 mM HEPES, 300 mM NaCl, 1.5 mM MgCl_2_, and 0.2 mM EDTA, pH = 7.9). The cell lysates were run on a SDS-PAGE gel, and blotted using the indicated antibodies. The densitometry of indicated blot was quantified using Image J software (National Institutes of Health, USA).

### Cytokine production assay

Concentrations of TNF-α, IL-6, IL-12p40, IL-10, GM-CSF, G-CSF, MIP-1α and MCP-1 were determined with commercially available Ready-Set-Go cytokine kits (eBioscience) in triplicate according to the manufacturer’s instructions.

### Murine peritoneal infection model

C57BL/6 female mice were injected intraperitoneally with 5 × 10^5^ UV-inactivated *C. albicans* and killed by CO_2_ asphyxiation after 4h. The inflammatory infiltrate was collected by lavage with ice-cold PBS containing 5 mM EDTA. Inflammatory cell populations were counted and then were analyzed by flow cytometry (BD FACS Verse^TM^) to determine the leukocyte composition as previously described^27^. Cytokines in peritoneal lavage fluid were determined as described above.

### Measurement of neutrophil killing of *C. albicans*

Thioglycollate-elicited peritoneal neutrophils (0.6 × 10^6^) were infected with live *C. albicans* (0.3 × 10^4^ to 1 × 10^4^) in the wells of a 24-well plate and were kept for 60 minutes at 4 °C to allow the cells to ‘settle’, before being transferred to an incubator at 37 °C for an additional 60 minutes. Cells were then mixed with scraping, and plated on YPD agar for counting viable *C. albicans* after incubation for 48h at 30 °C^27^.

### Respiration burst assay

Freshly isolated thioglycollate-elicited peritoneal neutrophils pooled from at least two mice, were loaded with 10 μM dihydrorhodamine 123, and then stimulated with *C. albicans* (MOI = 1) for 60 minutes at 37 °C. Reactive oxygen species production was determined by measuring the conversion of dihydrorhodamine 123 to rhodamine using a SpectraMax M5 multi-mode microplate reader with excitation and emission filters of 485 and 538 nm^51^.

### Statistical analysis

At least three biological replicates were performed for all experiments unless otherwise indicated. One-way analysis of variance (ANOVA) with Bonferroni or Dunns post-tests was used when multiple groups were analyzed. For analysis of nonparametrically distributed data, Kruskal-Wallis test was used. Statistical significance was set at *P* value of 0.05 or 0.01.

## Additional Information

**How to cite this article**: Liu, W. *et al*. Bst1 is required for *Candida albicans* infecting host via facilitating cell wall anchorage of Glycosylphosphatidyl inositol anchored proteins. *Sci. Rep*. **6**, 34854; doi: 10.1038/srep34854 (2016).

## Supplementary Material

Supplementary Information
