# Supplementary material for: Bst1 is required for Candida albicans infecting host via facilitating cell wall anchorage of Glycosylphosphatidyl inositol anchored proteins
Source: Sci Rep. 2016 Oct 6;6:34854. doi: 10.1038/srep34854 (PMC5052643; doi:10.1038/srep34854)
Supplement: Supplementary Information [file srep34854-s1.pdf]

Supplementary Information

Bst1 is required for *Candida albicans* infecting host via facilitating cell wall anchorage of Glycosylphosphatidyl inositol anchored proteins

Wei Liu[1†], Zui Zou[2 †], Xin Huang[3], Hui Shen[4], Li Juan He[5], Si Min Chen[1], Li Ping Li[1], Lan Yan[5], Shi Qun Zhang[1], Jun Dong Zhang[1], Zheng Xu[5], Guo Tong Xu[1], Mao Mao An[1*], Yuan Ying Jiang[1, 5*]

[1] Shanghai Tenth People's Hospital, and Department of Pharmacology, Tongji University School of Medicine, Shanghai, 200092, P.R. China
[2] Department of Anesthesiology, Changzheng Hospital, Second Military Medical University, Shanghai, 200433, P.R. China
[3] Department of dermatology, Shanghai Tongji Hospital, Tongji University School of Medicine, Shanghai, 200065, P.R. China
[4] Department of Laboratory Diagnosis, Changhai Hospital, Second Military Medical University, 168 Changhai Road, Shanghai 200433, P.R. China
[5] Research and Development Center of New Drug, School of Pharmacy, Second Military Medical University, Shanghai, 200092, P.R. China

* Corresponding author: jiangyy@tongji.edu.cn (YYJ); anmaomao@tongji.edu.cn (MMA)

[†] These authors contributed equally to this work.

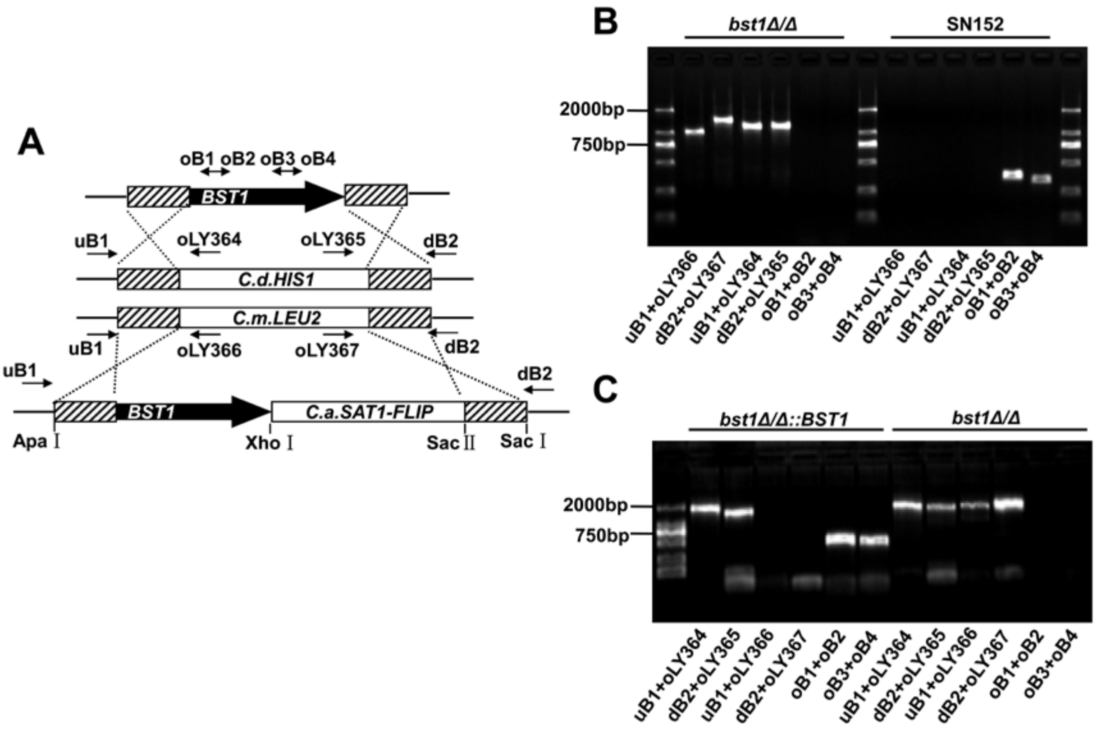

Supplementary Figure S1. Construction of *bst1* null mutant and the complemented strain.

(A) Two-step homologous recombination by a fusion-PCR-based strategy for *BST1* gene deletion and SAT1 flipping method for *BST1*-complemented strain construction from parent strain SN152. Arrows represent orientation and approximate position of primers used for PCR (Supplementary Table S2). **(B, C)** The genotype of C. albicans strains were confirmed by PCR. The genomic DNA of SN152, *bst1Δ/Δ* mutant and *BST1*-complemented strains were amplified with the indicated primers.

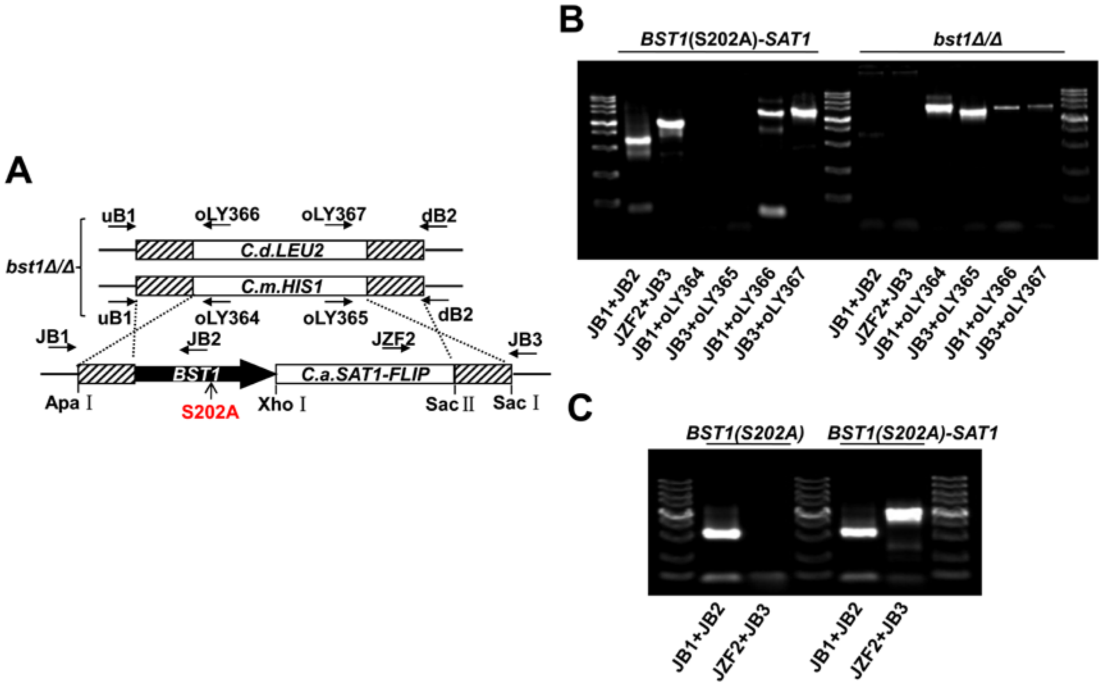

Supplementary Figure S2. Construction of *BST1* (S202A) mutant strain.

(A) SAT1 flipping method to substitute serine 202 of *BST1* gene by alanine in bst1Δ/Δ mutant strain. **(B, C)** The genotype of C. albicans strains were confirmed by PCR. The genomic DNA of *BST1* (S202A)-SAT1, *bst1Δ/Δ*, and *BST1 (S202A)* mutant strains were amplified with the indicated primers.

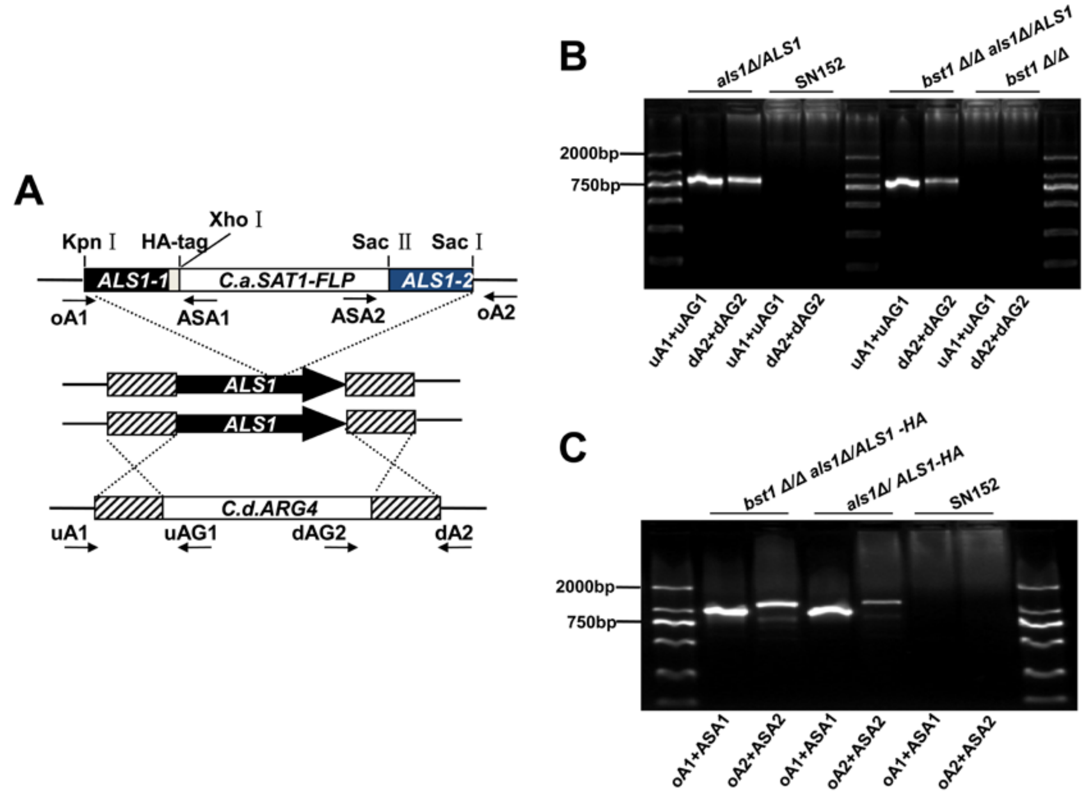

Supplementary Figure S3. Construction of C. albicans strains expressing hemagglutinin (HA)-tag fused Als1p.

(A) Homologous recombination by a fusion-PCR-based strategy for one allele of *ALS1* deletion and SAT1 flipping method for inserting HA-tag to another allele of ALS1 gene in *bst1Δ/Δ* mutant and parent strain SN152. **(B, C)** The genotype of C. albicans strains were confirmed by PCR. The genomic DNA of *als1Δ/ALS1*, SN152, *bst1Δ/Δ als1Δ/ALS1*, *bst1Δ/Δ*, *bst1Δ/Δ als1Δ/ALS1*-HA and *als1Δ/ALS1*-HA strains were amplified with the indicated primers.

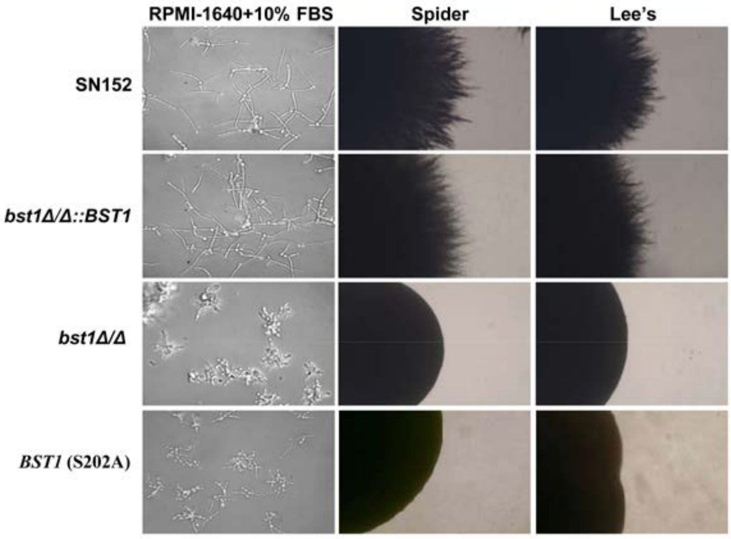

Supplementary Figure S4. Filamentation of *bst1* mutants was defective in different media favoring hyphal growth.

Exponentially growing C. albicans parent (SN152), *BST1*-complemented (*bst1Δ/Δ::BST1*), bst1 null mutant (*bst1Δ/Δ*), and *BST1* S202A mutant yeast cells were incubated in RPMI 1640 medium with 10% fetal bovine serum for 3 hours, or grew on spider and lee's agar media for 5 days at 37℃. Representative photographs were shown.

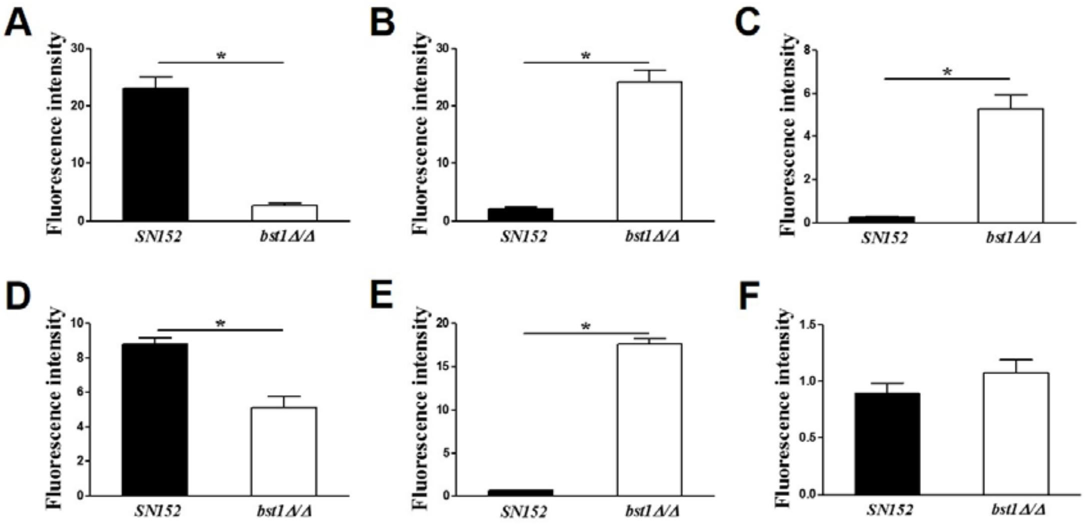

Supplementary Figure S5. Fluorescence intensity analysis of data shown in Figure 4B-G by the LAS AF Lite program (version 2.1.1, build 4443; Leica Microsystems).

(A-C). Fluorescence intensity of three cell wall carbohydrate layers from exponentially growing parent (SN152), *BST1*-complemented (*bst1Δ/Δ::BST1*), *bst1Δ/Δ* null mutant (*bst1Δ/Δ*), and *BST1* S202A strains, which were stained with ConA-FITC to visualize mannan (A), β-glucan antibody to visualize β-glucan (B) and CFW to visualize chitin (C).

(D-F). Fluorescence intensity of three cell wall carbohydrate layers from the hyphal forms of parent (SN152), *BST1*-complemented (*bst1Δ/Δ::BST1*), *bst1Δ/Δ* null mutant (*bst1Δ/Δ*), and *BST1* S202A mutant strains, which were stained with ConA-FITC to visualize mannan (D), β-glucan antibody to visualize β-glucan (E) and CFW to visualize chitin (F).

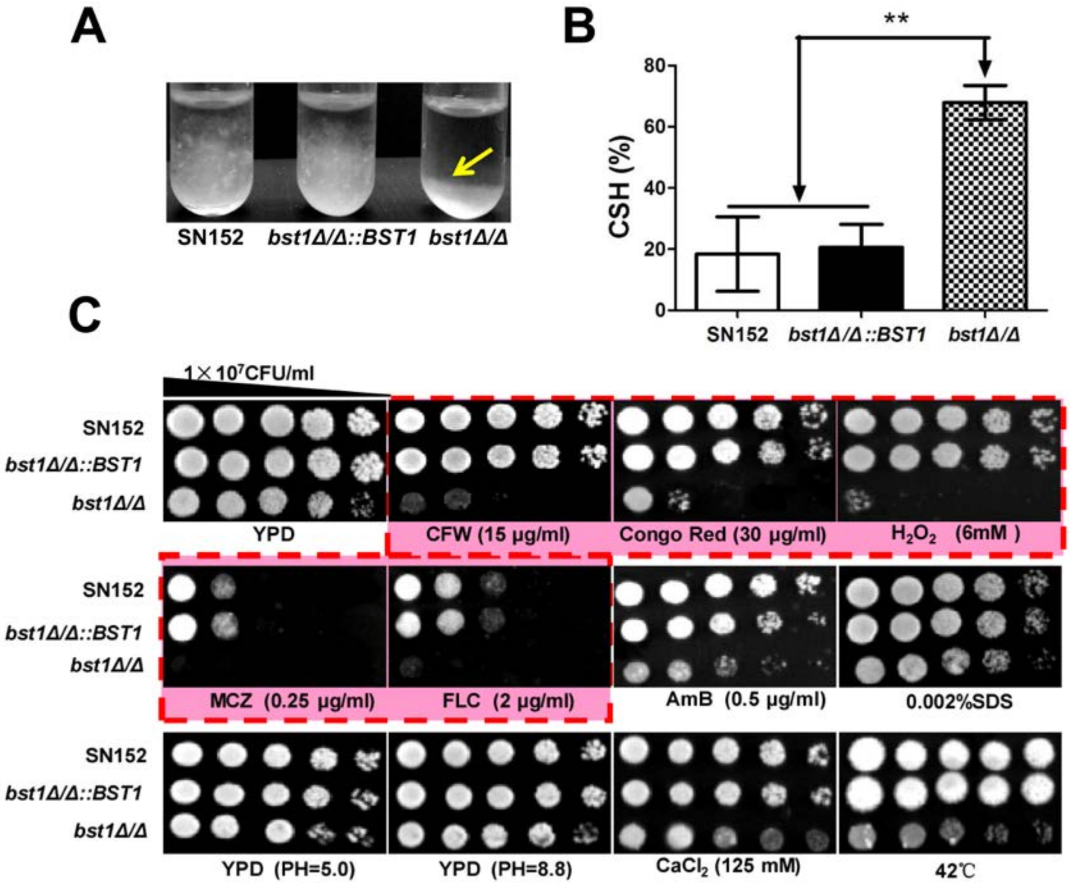

Supplementary Figure S6. Cell surface properties of C. albicans strains and their resistance to stress.

(A) Flocculation of parent (SN152), *BST1*-complemented (*bst1Δ/Δ*::*BST1*), *bst1* null mutant (*bst1Δ/Δ*) strains in RPMI 1640 medium for 10 minutes. Representative photographs were shown. **(B)** Cell surface hydrophobicity (CSH) of SN152, *bst1Δ/Δ::BST1*, and *bst1Δ/Δ* strains. The CSH was measured by the percentage of cells in the hydrophobic phase. **, $P<0.01$ (Error bars indicate SD. One-way ANOVA with Bonferroni post-test). **(C)** Resistance of SN152, *bst1Δ/Δ::BST1*, and *bst1Δ/Δ* strains to various indicated stresses. Serial 5-fold dilutions of the C. albicans strains were inoculated on YPD plates supplemented with different stresses.

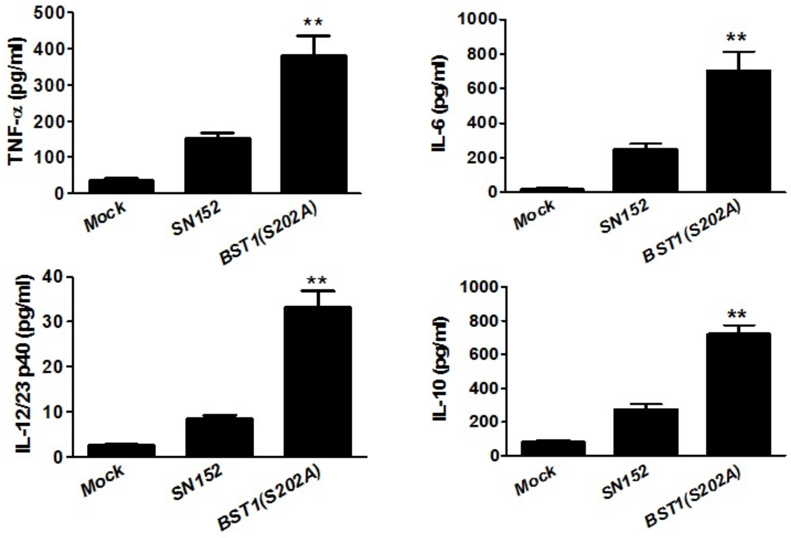

Supplementary Figure S7. Inflammatory responses in macrophage stimulated by *C. albicans BST1* (S202A) mutant.

Thioglycollate-elicited peritoneal macrophages were stimulated with parent strain SN152, *BST1* (S202A) mutant (MOI=5) for 6 hours. TNF-α, IL-6, IL-12p40 and IL-10 in supernatants of thioglycollate-elicited peritoneal macrophages were measured by ELISA. Mock, unstimulated macrophages. Data represent mean (±SD) of triplicates of one representive experiment of three. **, P <0.01 (Two-way ANOVA with Bonferroni post-test).

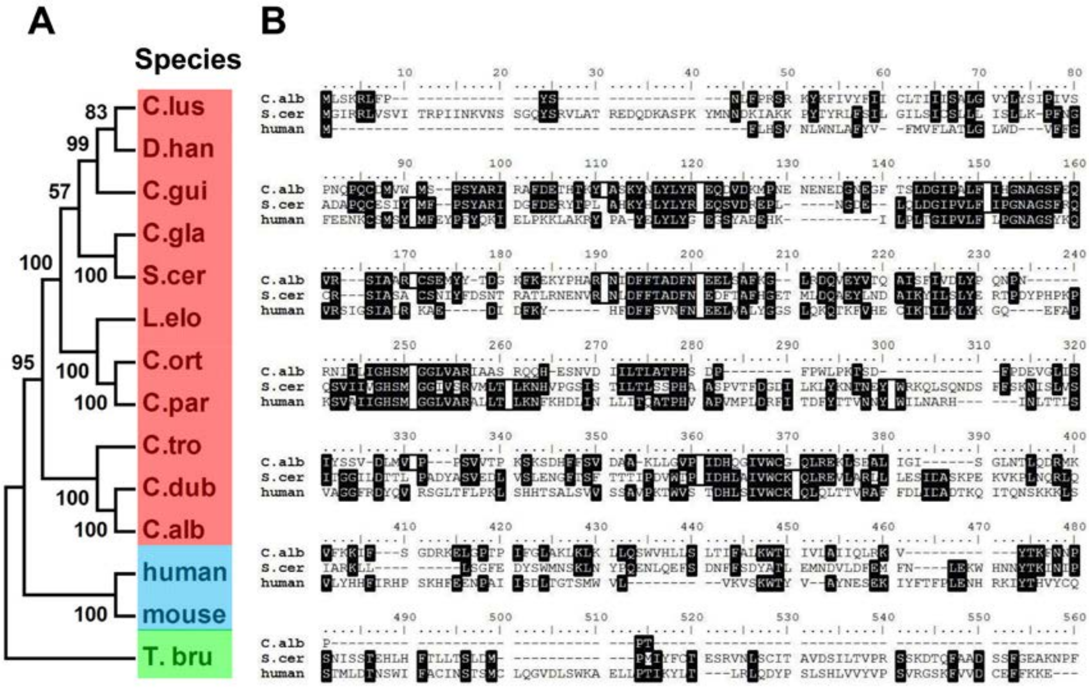

Supplementary Figure S8. The molecular phylogeny and alignment of inositol deacylase in different species.

(A) Fourteen homologous inositol deacylase in different species are displayed according to the phylogenic tree representation. The conserved domains of sequences was analyzed by the MEGA4 program. **(B)** Multi-protein sequence alignment highlighting the protein differences between C. albicans and mammalian cells.

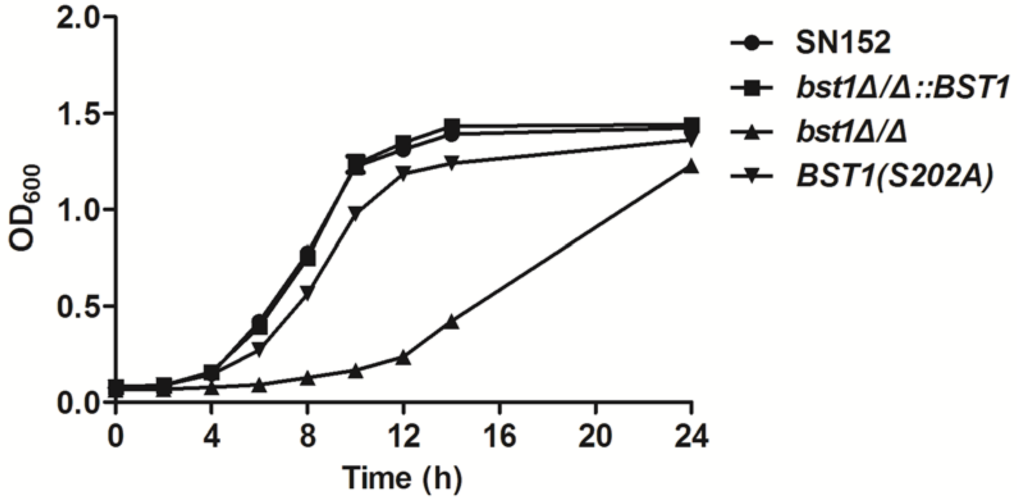

Supplementary Figure S9. Growth curves of *bst1* null mutant (*bst1Δ/Δ*) and *BST1* S202A mutant strains.

Supplementary Table S1. ***C. albicans*** **strains and plasmids used in this study**

Strain	Relevant genotype	reference
C. albicans		
SN152	*arg4Δ /arg4Δ leu2Δ/leu2Δ his1Δ/his1Δ URA3/ura3Δ::imm*^{434} *IRO1/iro1Δ ::imm* ^{434}	Noble et al., 2005
bst1Δ /Δ	*bst1Δ::HIS1/ bst1 Δ::LEU2 arg4 Δ /arg4Δ URA3/ura3Δ::imm*^{434} *IRO1/iro1Δ ::imm* ^{434}	This study
bst1Δ/Δ::BST1	*bst1Δ::HIS1/ bst1 Δ::BST1::SAT1-FLIP arg4Δ /arg4Δ URA3/ura3Δ::imm*^{434} *IRO1/iro1Δ ::imm* ^{434}	This study
als1Δ/ALS1	*als1Δ::ARG4 / ALS1 leu2Δ/leu2Δ his1Δ/his1Δ URA3/ura3Δ::imm*^{434} *IRO1/iro1Δ ::imm* ^{434}	This study
bst1Δ /Δ als1Δ/ALS1-HA	*bst1Δ::HIS1/ bst1 Δ::LEU2 ALS1::HA::SAT1-FLIP / als1Δ::ARG4 URA3/ura3Δ::imm*^{434} *IRO1/iro1Δ ::imm* ^{434}	This study
BST1 (*S202A*)	*bst1Δ::LEU2/ bst1 Δ::BST1(S202A)::SAT1-FLIP arg4Δ /arg4Δ URA3/ura3Δ::imm*^{434} *IRO1/iro1Δ ::imm* ^{434}	This study
**Plasmid**		
pSN40	*C. maltosa LEU2*	Noble et al., 2005
pSN52	*C. dubliniensis HIS1*	Noble et al., 2005
pSN69	*C. dubliniensis ARG4*	Noble et al., 2005
pSFS2	*FRT-MAL2p-CaFLP-ACT1-CaSAT1-FRT* into pSFI1	Reuss et al., 2004

Supplementary Table S2. Primers used in this study

Primer	Sequence	Application
oB1	ATGCCCAACGAGAATGAAAA	*BST1* specific
oB2	CTTGAAGCCGCAATTCTAGC	*BST1* specific
oB3	TGGCAACACCACATTCTGAT	*BST1* specific
oB4	TGGTGTTGGACCCAATTCTT	*BST1* specific
uB1	CCCCAATCCACTACTTGTCC	*BST1* upstream Check
dB2	AAAAACCGCTTCCACTGATG	*BST1* downstream Check
oLY364	TCAAGCCCTGTAGCTCCATT	*HIS1* check
oLY365	TCCGCTCATTTGATTTCCTC	*HIS1* check
oLY366	GCACGCCGTTACAGGAGTTA	*LEU2* check
oLY367	GAAGTTGGTGACGCGATTGT	*LEU2* check
P2	ccgctgctaggcgcgccgtgACCAGTGTGATGGATATCTGC (Noble et al., 2005)	for *HIS1*, *LEU2*, and *ARG4* cassettes
P5	ccagggatgcggccgctgacAGCTCGGATCCACTAGTAACG (Noble et al., 2005)	for *HIS1*, *LEU2*, and *ARG4* cassettes
BST1 P1	TGTATTTCCCCCATGGTGTT	Upstream region of *BST1* for disruption
BST1 P3	cacggcgcgcctagcagcggATGGTTGGGTGGCTTTAGTG	Upstream region of *BST1* for disruption
BST1 P4	gtcagcggccgcatccctgcAACCCTCCACCCACACACTA	Downstream region of *BST1* for disruption
BST1 P6	CCAGGTGAATTGGCTAAACA	Downstream region of *BST1* for disruption
BST1 huF	GCC GGGCCC TCCCCAGATATTTCTCCTTTTTC	Upstream region of *BST1* with Apa I restriction sequence to reconstituted *BST1*
BST1 huR	GCC CTCGAG CTAGTGTGTGGGTGGAGGGT	with Xho I

		restriction sequence to reconstituted *BST1*
BST1 hdF	GCC CCGCGG AACCCTCCACCCACACACTA	Downstream region of *BST1* with Sac II restriction sequence to reconstituted *BST1*
BST1 hdR	GCC GAGCTC TTTTGAAACAAACTCACCCAGA	Downstream region of *BST1* with Sac I restriction sequence to reconstituted *BST1*
JB1	CCACCAAGGAAAGCTGGTAG	*BST1* upstream Check
JB3	GCTTCCACTGATGGTGCTAA	*BST1* downstream Check
JB2	ATGGTTGGGTGGCTTTAGTG	*BST1* Check
BST1(S202A) R1	AGTCCACCCATTGCATGACC	*BST1*with S202A mutation
BST1(S202A) F2	GGTCATGCAATGGGTGGACT	*BST1*with S202A mutation
uA1	TTGCAAAAATTTCCAACTGGT	*ALS1* upstream Check for one allele disruption
dA2	CAACTTGCTTTGCTCTGCAC	*ALS1* downstream Check for one allele disruption
ARG4 JR	CTCAATAAAACAATCCTGGGT	*ARG4* check
ARG4 JF	TTCAAAATAGGTATCACGGGC	*ARG4* check
ALS1 uF	GCC GGTACC AGCACAACCTCCGATTTGTC	One region of *ALS1* with Kpn I restriction sequence to add HA-tag into *ALS1*
ALS1 uR	TTGGTTGTCTCCATTGCTTG	One region of

		ALS1 to add HA-tag into *ALS1*
ALS1 HA F	CAAGCAATGGAGACAACCAA atcttttacccatacgatgttcct	One region of HA tag add HA-tag into *ALS1*
ALS1 HA R	GCC CTCGAG ctgagcagcgtaatctggaa	One region of HA tag with Xho I restriction sequence to add HA-tag into *ALS1*
ALS1 dF	GCC CCGCGG AGTGGTACTCATGATTCACAATCT	One region of ALS1 with Sac II restriction sequence to add HA-tag into *ALS1*
ALS1 dR	GCC GAGCTC TGGAGATTTACCACCATTTTCA	One region of *ALS1* with Sac I restriction sequence to add HA-tag into ALS1
oA1	ATTTGCCACAACCACCACAG	Check for HA-tagged *ALS1*
oA2	TCATTTGAAGCACTGGCAAC	Check for HA-tagged *ALS1*
JZR1	GCACGCTAGACAAATTCTTCC	Check for HA-tagged *ALS1*
JZF2	CAGCTCCTTGGCATACGATT	Check for HA-tagged *ALS1*

Supplementary Table S3. Twenty one GPI-APs with significantly impaired cell wall anchorage in *BST1*-deficient *C. albicans* strains

Proteins	Function	Relative fold change (*bst1Δ/Δ* versus parent strain)
Pga4	1,3-beta-glucanosyltransferase	0.012
Cht2	Hydrolysis of N-acetyl-beta-D-glucosaminide (1->4)-beta-linkages in chitin and chitodextrins.	0.009
Utr2	Extracellular glycosidase	0.036
Dfg5	Endo-1,6-alpha-D-mannosidase	0.078
Ecm33	Cell wall integrity and for the correct assembly of the mannoprotein	0.014
Dcw1	Endo-1,6-alpha-D-mannosidase	0
Ccw14	Cell wall structure	0.009
Ihd1	Cell wall organization, hyphal growth	0
Crh11	Extracellular glycosidase.	0.038
Als1	Mediates both yeast-to-host tissue adherence and yeast aggregation	0.013
Pga24/Ywp1	Anti-adhesive and promotes dispersal of yeast forms	0.283
Pga29/Rhd3	Hyphae development	0.049
Sod4	Cell surface superoxide dismutase [Cu-Zn] 4	0
Sod5	Cell surface superoxide dismutase [Cu-Zn] 5	0.047
Plb5	Phospholipase activity	0.147
Carp9	Secreted aspartic peptidases	0
Csa	Heme-binding	0.024
Rbt5	Heme-iron utilization	0.005
Pga52	Unknown	0.123
Pga30	Unknown	0.015
Pga45	Unknown	0
Kre9**	Beta-1,6-glucan biosynthesis	0.740

Kre9*, a cell wall non-GPI anchored protein was shown as a control.

Supplemental Experimental Procedures

Strain construction

The C. albicans strains and plasmids used in these studies and their relevant genotypes are listed in Supplementary Table S1. All strains were maintained on SDA agar (1% peptone, 4% dextrose, and 1.8% agar) plates and re-cultured at least monthly from -80℃ stock. For use in the experiments, yeast-phase cells of the various strains were grown in YPD broth overnight in a rotary shaker at 30℃.

Construction of *CaBST1*-deleted and complemented strains (Supplementary Fig. S1). *CaBST1* gene was knocked out in the parental strain SN152 by using a two-step homologous recombination approach based on a fusion-PCR-based strategy[1] (Supplementary Fig. S1). Plasmid pSN52 and pSN40 were used as templates to amplify the auxotrophic markers *Candida dubliniensis* HIS1 or *Candida maltosa* LEU2 by PCR with primer P2 and P5. 3' and 5'flanking homologous sequences were amplified by PCR with primer pairs *BST1*P1-*BST1*P3 and *BST1*P4-BST1 P6 (Supplementary Table S2), using strain SN152 genomic DNA as templates. The knockout cassettes (*bst1Δ::C.d.*HIS1 and *bst1Δ::C.m.*LEU2) containing auxotrophic markers and homologous sequences were generated by fusion-PCR with primers *BST1*P1 and *BST1*P6 (Table S2). PCR products were used to transform parent strain SN152 using yeast transformation II kit (Clotech) following the manufacturer's protocol and selected on synthetic defined (SD) medium (2% glucose, 0.67% yeast nitrogen base and 0.2% amino acid mix) lacking histidine for the first allele deletion

of the *CaBST1*, and lacking both histidine and leucine for the second allele deletion of the *CaBST1*. To construct the *BST1*-complemented strain (*bst1Δ/Δ::BST1*), ApaI-XhoI fragment containing 5' homologous sequence and the entire *BST1* coding sequence, and SacII-SacI fragment containing 3' homologous fragment were cloned into the plasmid pSFS2A, respectively[2]. The resulting plasmid, containing SAT1-flipper cassette was linearized by digestion with SacI and transformed into *bst1Δ/Δ* using yeast transformation II kit (Clotech), as described by the manufacturer, and selected on YPD agar (1% yeast extract, 2% peptone, 2% dextrose, and 1.8% agar) plates with 200 μg/ml nourseothricin. The genotype of *bst1* null mutant (*bst1Δ/Δ*) and *BST1*-complemented strains were confirmed by PCR with primers uB1-oLY366, uB1-oLY364, dB2-oLY365, dB2-oLY367, oB1-oB2 and oB3-oB4 listed in Table S2 (Supplementary Fig. S1B and C).

BST1 S202A mutant was constructed in *bst1Δ/Δ* mutant strain (Supplementary Fig. S2A). ApaI-XhoI fragment containing 5' homologous sequences and the *BST1* coding sequence with the putative catalytic serine 202 substituted by alanine, and SacII-SacI fragment containing 3' homologous fragment were cloned into the plasmid pSFS2A, respectively[2]. The resulting plasmid, containing SAT1-flipper cassette was linearized by digestion with SacI and transformed into *bst1Δ/Δ* using yeast transformation II kit (Clotech) following the manufacturer's protocol and selected on YPD agar plates with 200 μg/ml nourseothricin. The genotype of *BST1*(S202A) mutants were confirmed by PCR with primers JB1-oLY366, JB1-oLY364, JB3-oLY365, JB3-oLY367, JB1- JB2 and JZF2-JB3 listed in Supplementary Table S2 (Supplementary Fig. S2B and C).

Strains expressed hemagglutinin (HA)-tag fused Als1p (*bst1Δ/Δ als1Δ/ALS1*-HA and *als1Δ/ALS1*-HA) were generated in strain *bst1Δ/Δ* and parent strain SN152 (Supplementary Fig. S3A). One allele of *ALS1* was replaced by auxotrophic markers C. dubliniensis ARG4 by homologous recombination approach based on a fusion-PCR-based strategy as described above. A 102 bp fragment containing three repeated sequence of hemagglutinin (HA)-tag was inserted into the ALS1 gene allele by SAT1 flipping method. The resulting plasmid was linearized by digestion with SacI and transformed into the above strain *bst1Δ/Δ* and SN152 with one allele of ALS1 deletion. The genotype of *bst1Δ/Δ als1Δ*/*ALS1*-HA and *als1Δ/ALS1*-HA strains were confirmed by PCR with primers uA1-uAG1, dA2-dAG2, oA1-ASA1 and oA2-ASA2 listed in Supplementary Table S2 (Supplementary Fig S3B and C).

Supplementary Reference List:

1 Noble, S. M. & Johnson, A. D. Strains and strategies for large-scale gene deletion studies of the diploid human fungal pathogen *Candida albicans*. *Eukaryotic cell* **4**, 298-309, doi:10.1128/EC.4.2.298-309.2005 (2005).

2 Reuss, O., Vik, A., Kolter, R. & Morschhauser, J. The SAT1 flipper, an optimized tool for gene disruption in *Candida albicans*. *Gene* **341**, 119-127, doi:10.1016/j.gene.2004.06.021 (2004).
